# Neurological manifestations and neuroimaging findings in patients with SARS-CoV2—a systematic review

**DOI:** 10.1186/s41983-021-00322-3

**Published:** 2021-06-02

**Authors:** Nikita Mohan, Muhammad Ali Fayyaz, Christopher del Rio, Navpreet Kaur Rajinder Singh Khurana, Sampada Sandip Vaidya, Esteban Salazar, John Joyce, Amrat Ayaz Ali

**Affiliations:** 1 Jawaharlal Nehru Medical College, Belagavi, Karnataka India; 2 Quaid e Azam Medical College, Bahawalpur, Pakistan; 3 Tecnologico de Monterrey, Escuela de Medicina y Ciencias de la Salud, Monterrey Nuevo Leon, Mexico; 4 Indira Gandhi Government Medical College & Mayo Hospital, Nagpur, Maharashtra India; 5 Kazan State Medical University, Kazan, Russia; 6 M.S Ramaiah Medical College, Bangalore, Karnataka India; 7 Liaquat University of Medical and Health Sciences, Jamshoro, Pakistan

**Keywords:** SARS-CoV 2, Stroke, Neuro-invasive, COVID-19, Neuroimaging

## Abstract

**Background:**

The COVID-19 pandemic has drastically affected everyone in a hit or miss manner. Since it began, evidence of the neuro-invasive potential of the virus has been intensifying significantly. Several pathways have been hypothesized to elucidate the neurotropic nature of SARS-CoV2. It is the need of the hour to collect vital information.

**Objective:**

To evaluate and correlate the neuro-radiological and neurological manifestations in patients diagnosed with SARS-CoV2.

To identify neuro-invasive pathways of COVID infection.

**Methods:**

Relevant studies were identified through four databases—the Cochrane Library, PubMed, Science Direct, and Web of Science. These were searched using relevant keywords—“COVID-19,” “SARS-CoV2,” “neurological manifestations,” “neuroimaging,” “CT,” and “MRI.” Relevant articles were screened according to a pre-defined inclusion and exclusion criteria from December 2019 to August 2020.

**Results:**

Our review included a total of 63 full text publications with 584 patients, composed mainly of observational studies, case reports, and case series. The most common neurological manifestations associated with COVID-19 were altered mental status, stroke, and paralysis. About 17.85% patients who underwent neuroimaging were found to be having ischemic changes suggestive of a stroke. This was followed by hemorrhagic changes as the second most common finding. The most commonly involved vessel was the Middle Cerebral Artery. Besides stroke, we found that SARS-CoV2 could be the cause for new-onset seizures, Guillain-Barre Syndrome, encephalitis, and many other severe neurological diseases.

**Conclusion:**

The information that we have obtained so far will prove dynamic to healthcare providers working against the COVID-19 pandemic. It is necessary to be aware of these atypical neurological findings for the early diagnosis and treatment of COVID-19 infected patients. However, to completely understand the connection between SARS-CoV2 and the nervous system, further research is necessary.

## Introduction

The infamous COVID-19 pandemic has drastically involved everyone in a hit or miss manner. The world is currently fighting against a highly infectious novel coronavirus, known as SARS-CoV2. What began as an outbreak of pneumonia in Wuhan, China, has rapidly engulfed the entire world [1]. As of August 31, 2020, this virus has infected approximately 25 million people and caused 844 thousand deaths globally [2]. The pandemic has posed severe challenges to public health, and the medical community continues to struggle in hitherto mysterious zones, especially in terms of reliable therapeutic interventions. In one study, health care providers utilized extracorporeal membrane oxygenation (ECMO) for patients with acute respiratory distress syndrome secondary to COVID-19, although early reports seem to have a high mortality rate due to devastating neurological insult [3].

Though the respiratory symptoms are the most common, there have been studies which highlight the potential neurotropism of the virus. The incubation period of COVID-19 infected patients, whether asymptomatic or possessing wide spread signs and symptoms, varies from 2 to 11 days with an approximate mortality rate of 2-4% [4]. In an observational study in Wuhan, 36.4% of the patients had neurological involvement such as impaired consciousness, acute cerebrovascular events, headache, seizure, hyposmia, and hypogeusia [5]. There have also been several reports on patients presenting with neurological involvement as the initial symptoms [6, 7].

This initial data reflects that the brain seems to be a target organ for various infections and critical diseases, either due to direct insult or through secondary involvement. The peripheral nervous system (PNS) is also particularly susceptible during infection-related immune-mediated diseases [8].

Even though there is extensive data on the respiratory involvement of SARS-CoV2, documentation of its neurological aspect has been limited to observational studies and case reports. There is a further lack of information on the neuroimaging findings of COVID-19. In this rapidly evolving situation, it has become essential for healthcare providers to stay updated on the various atypical presentations of SARS-CoV2 and keep in mind COVID-19 as a potential diagnosis when encountering such cases. Therefore, we performed a comprehensive literature search in this systematic review to ascertain the different neurological manifestations and neuroimaging findings linked with COVID-19 infection.

## Objective

To evaluate and correlate the neuro-radiological and neurological manifestations in patients diagnosed with SARS-CoV2.

To identify neuro-invasive pathways of COVID infection.

## Methods

A comprehensive search of the literature was performed from the following databases: PubMed, Web of Science, Cochrane Library, and Science Direct. The following search terms were used in combination with the Boolean operators AND and OR; “COVID-19,” “SARS-CoV2,” “neurological manifestations,” “neuroimaging,” “MRI,” and “CT.” We selected for analysis only articles in which the title and abstract contained the aforementioned search terms. In an initial screen, we excluded articles which were duplicates, and those in which title and abstract were not relevant to our search terminology. Of the remaining studies, screening was done based on the full text of the article under the following inclusion criteria: (1) Studies reporting patients with laboratory confirmation of SARS-CoV2, (2) case reports, case series, cohort studies, and case-control studies, (3) studies in which subjects were above the age of 18, (4) studies containing neuroimaging (CT or MRI) of the brain, (5) studies performed between December 2019 and August 2020. The exclusion criteria were as follows: (1) reviews, editorials, or commentaries. (2) Studies in which subjects were in the pediatric age group, were pregnant, or had prior neurological conditions. (3) Studies with no neurological evaluation, (4) studies published in any language other than English, without available English translations. The articles were screened in their entirety, by two independent readers, in each of the aforementioned scientific databases, to determine eligibility for inclusion. Discrepancies were discussed among all authors, and a collective effort was undertaken to resolve them.

**Fig. 1:**
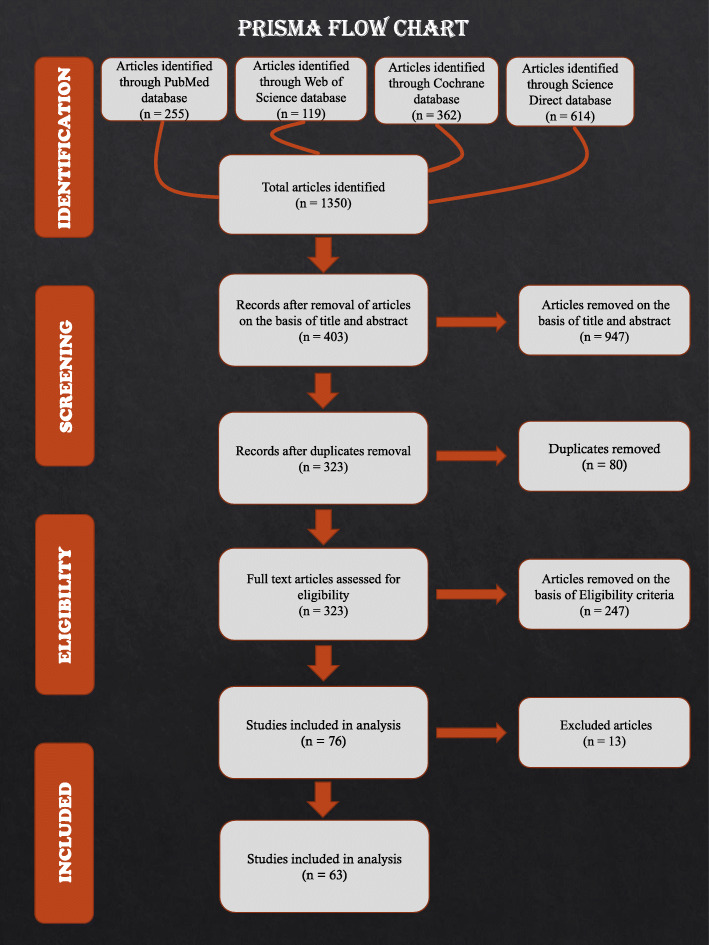
PRSIMA flow chart summarizing search strategy for the articles included in the study

The search strategy and article selection process are depicted in the flowchart in Fig. 1 as per the PRISMA statement.

## Results

Through the search strategy, we identified 63 articles with neurological and neuroimaging manifestations in patients infected with COVID-19. We included 584 patients who presented with neurological manifestations and underwent different neuroimaging modalities. The age of patients ranged from 24-88 years.

**Fig. 2:**
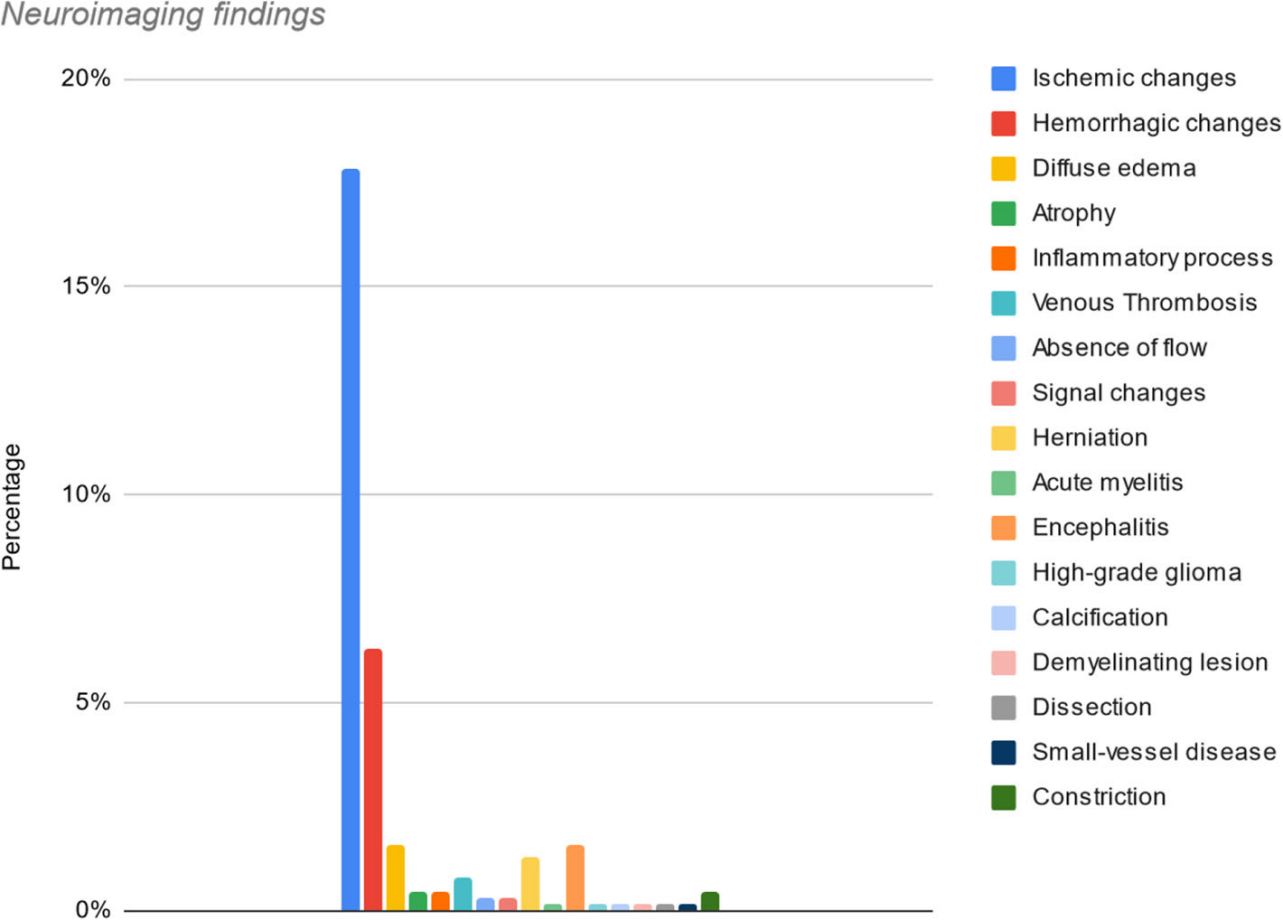
Evaluation of positive neurological findings on CT scan and MRI of COVID-19 infected patients. To demonstrate positive neuroimaging findings, patients with normal findings on imaging or findings unrelated to COVID-19 were not included

In terms of neuroimaging findings (Table 1), among these 63 articles, 584 patients underwent neuroimaging. Four hundred and twenty eight (67.61%) patients that underwent neuroimaging did not have any abnormality on CT or MRI. For the remaining 156 patients, neuroimaging findings were in descending order as follows: ischemic changes (17.85%), with the middle cerebral artery (MCA) being the most frequent anatomical location; hemorrhagic changes (6.31%), diffuse edema (1.57%), encephalitis (1.57%), herniation (with uncal and subfalcine as the most common) (1.26%), venous thrombosis (0.7%), atrophy (0.4%), inflammatory process (0.4%), and constriction (0.4%). The absence of flow and signal changes was 0.3% each. The least common findings were acute myelitis, high-grade glioma, calcification of the proximal left internal carotid artery (ICA), a demyelinating lesion in left temporal and right occipital lobes, dissection of the left vertebral artery, and small-vessel disease comprised the remaining 0.6% (0.1% each) (Fig. 2).

**Table 1:** Reported studies on COVID-19 patients with neurological manifestations with positive findings on major imaging modalities

	Article name	Imaging modality	Neuroimaging findings	
1	A case of COVID-19 respiratory Illness with Subsequent seizure and hemiparesis [9]	CT—head	Subcortical hypoattenuation with sulcal effacement in the left occipital and posterior parietal lobes suggestive of ischemic changes	
2	A case series of devastating intracranial hemorrhage during venovenous extracorporeal membrane oxygenation for COVID-19 [3]	CT—head	Multicompartment intracranial hemorrhage with marked diffuse edema and secondary infarction of the left anterior and posterior cerebral artery territories due to vascular compression Multifocal intracerebral hemorrhage (ICH) with left hemispheric lobar hemorrhage and right cerebellar hemorrhage Small left frontal cortical subarachnoid hemorrhage (SAH)	
3	A first case of meningitis/encephalitis associated with SARS-coronavirus-2 [10]	MRI—brain	Diffusion weighted images (DWI) showed hyperintensity along the wall of inferior horn of right lateral ventricle. Fluid-attenuated inversion recovery (FLAIR) images showed hyperintense signal changes in the right mesial temporal lobe and hippocampus—suggestive of right lateral ventriculitis and encephalitis.	
4	Acute abducens nerve palsy in a patient with the novel coronavirus disease (COVID-19) [11]	MRI—brain	Denervation of CN VI- as evident by hyperintensity on T2 weighting of atrophic left lateral rectus muscle	
5	Acute disseminated encephalomyelitis after SARS-CoV-2 infection [12]	MRI—brain and spine	6 enhancing lesions, most with ring enhancement and some with nodular enhancement Hyperintense signal of the optic nerves bilaterally Hyperintense spindle-like T8 lesion	
6	Acute myelitis as a neurological complication of COVID-19: a case report and MRI findings [13]	Gadolinium-enhanced MRI—spine	Extensive diffuse hyperintense signal of the gray matter of cervical, dorsal, and lumbar regions of the spinal cord Mild enlargement and swelling of the cervical cord Areas of restricted diffusion on DWI and apparent diffusion coefficient (ADC)	
7	Acute polyradiculoneuritis with locked-in syndrome in a patient with COVID-19 [14]	MRI—spine	Massive symmetrical contrast enhancement of the spinal nerve roots at all levels of the spine including the cauda equina	
8	Acute profound sensorineural hearing loss after COVID-19 pneumonia [15]	MRI—brain	Pronounced contrast enhancement in the right cochlea and a partially decreased fluid signal in the basal turn of the right cochlea Adjacent to the temporal bone, meningeal contrast enhancement was seen at the base of the right temporal lobe Signs of an inflammatory process in the cochlea	
9	Basal ganglia involvement and altered mental status: a unique neurological manifestation of coronavirus disease 2019 [16]	CT—head MRI—brain	B/L basal ganglia hyper-density suggestive of subacute hemorrhagic event Involvement of basal ganglia in subacute bleeding	
10	Bilateral posterior cerebral artery territory infarction in a SARS-Cov-2 infected patient: discussion about an unusual case [17]	MRI—brain	B/L and asymmetric acute occipito-temporal infarction of the posterior cerebral arteries (PCA) with occlusion of P3 segments Hemorrhagic transformation of the previous lesions	
11	Bilateral trochlear nerve palsy due to cerebral vasculitis related to COVID-19 infection [18]	MRI—brain	Signs of vasculitis of the vertebrobasilar system Inflammatory signs in the periaqueductal region, along the topography of the trochlear nuclei	
12	Cerebral microhemorrhage and purpuric rash in COVID-19: the case for a secondary microangiopathy [19]	MRI—brain	Multiple areas of micro-hemorrhage throughout the corpus callosum, B/L juxtacortical white matter, basal ganglia, cerebellum, and brain- stem, without clear asymmetry Discrete areas of FLAIR hyperintensity correlating with some of the larger areas of SWI changes suggesting larger macro-hemorrhage Areas of diffusion restriction	
13	Cerebral nervous system vasculitis in a COVID-19 patient with pneumonia [20]	CT—headMRI—brain	Cortical-subcortical blood-related hyperdensities in the right occipital lobes and B/L fronto-parietal Signal restriction of the cortex in a parietal and parieto-occipital region and at the pons level suggestive of subacute phase of cortical inflammation and ischemia	
14	Cerebral venous thrombosis: a typical presentation of COVID-19 in the young [21]	CT—head MRI—brain	Left temporoparietal hemorrhagic venous infarct with edema and mass effect with 5 mm rightward shift Hyperintense DWI signal of the left temporoparietal hemorrhagic infarct with mass effect and effacement of the left lateral and third ventricle with 4 mm rightward shift Absence of flow in the sigmoid sinus, left transverse and internal jugular vein (IJV) secondary to venous thrombosis	
15	Coexistence of COVID-19 and acute ischemic stroke report of four cases [22]	MRI—brain	Total middle cerebral artery (MCA) infarction Left lenticulostriate artery infarction Right pontine infarction	
16	Concomitant neurological symptoms observed in a patient diagnosed with coronavirus disease 2019 [23]	CT—head	No abnormality	
17	Coronavirus 2019 (COVID-19)-associated encephalopathies and cerebrovascular disease: the New Orleans experience [24]	CT—head MRI—brain	Focal encephalitides and vasculolitides Diffuse hypoattenuation, focal hypodensities in deep structures, subacute ischemic strokes, and subcortical parenchymal hemorrhages Viral encephalitis: restriction and FLAIR changes in corpus callosum as well as B/L deep structures	
18	COVID-19 presenting as stroke [25]	CT—head CTA MRI—brain	Case 1—Loss of gray-white differentiation at the left occipital and parietal lobes, consistent with acute infarct. Evolution of a large acute infarct in the left MCA territory with hyperdense appearance of left MCA vessels—consistent with an acute thrombus Case 2—Moderate hypodensity in the right frontal lobe suggestive of an acute infarct Case 3—Occlusion of the right internal carotid artery (ICA) at origin with a core infarct in the right MCA distribution and a surrounding ischemic penumbra Case 4—acute infarct in the left medial temporal lobe Chronic microvascular ischemic changes Acute left MCA infarct Multiple small acute infarcts in B/L cerebral hemispheres Large acute hemorrhage in the brainstem and right cerebral hemisphere Ischemic and hemorrhagic stroke, hypoxic anoxic brain injury, encephalitis Severe cerebral edema with mass effect, diffuse cerebral sulcal effacement, brainstem compression with narrowing of the 4th ventricle due to downward cerebellar tonsillar herniation Severe diffuse cerebral arterial and dural venous sinus constriction	
19	COVID-19 presenting with seizures [26]	CT—head		
20	COVID-19 related neuroimaging findings: a signal of thromboembolic complications and a strong prognostic marker of poor patient outcome [27]	CT—head		
21	COVID-19-associated encephalopathy with fulminant cerebral vasoconstriction: CT and MRI findings [28]	CT—Head MRI MRA MRV		
22	COVID-19-associated encephalopathy: neurological manifestation of COVID-19 [29]	CT—head MRI—brain	Hypodensity of bilateral thalami Signal changes of brain parenchyma including insula, B/L dorsal frontal lobes, and thalamus with restricted diffusion of globus pallidus (features of encephalopathy)	
23	COVID-19-associated ophthalmoparesis and hypothalamic involvement [30]	MRI—brain	T2/FLAIR Hyperintensity (HI) in the brainstem, including the medial temporal lobes, mammillary bodies, CN VI nuclei, thalami, and hypothalamus	
24	COVID-19-associated pulmonary and cerebral thromboembolic disease [31]	CT—head MRI—brain	Partial right Sylvian segment (M2), superior division occlusion and right opercular (M3), parietal segment occlusions Multiple, discrete, peripheral acute infarctions of the right MCA territory with some hemorrhagic conversion	
25	COVID-19-related acute necrotizing encephalopathy with brain stem involvement in a patient with aplastic anemia [32]	CT—head MRI—brain	Increased hypodensity and swelling of the brain stem, and a new area of cortical and subcortical hypodensity in the left occipital lobe suggestive of an acute posterior circulation infarct Extensive, symmetrical changes in the supratentorial and infratentorial compartments. Hemorrhage and diffuse swelling in the amygdalae and brain stem Microhemorrhage and extensive abnormal signal were found in a symmetrical distribution within the dorsolateral putamina, ventrolateral thalamic nuclei, sub-insular regions, splenium of the corpus callosum, cingulate gyri, and subcortical perirolandic regions	
26	COVID-19-related strokes in adults below 55 years of age: a case series [33]	CT—head	Right MCA, Left MCA, and left basal ganglia infarction	
27	COVID-19-associated encephalitis mimicking glial tumor [34]	MRI—brain	Hyperintense signal in the left temporal lobe in T2 and T2 FLAIR imaging suggestive of high-grade glioma	
28	De novo status epilepticus in patients with COVID-19 [35]	CT—head MRI—brain	No abnormality	
29	Delirium as a presenting feature in COVID-19: neuroinvasive infection or autoimmune encephalopathy? [36]	CT—head MRI—brain	Case 1—3 hyperintense foci on diffusion suggesting cellular infiltration/inflammation or small infarcts Case 2—Changes in the limbic system with partial diffusion restriction, consistent with limbic encephalitis	
30	Emergency room neurology in times of COVID-19: malignant ischaemic stroke and SARS-CoV-2 infection [7]	CT—head CTA	Established infarct in the territory of the left MCA with a mild deviation of the midline Occlusion of the left MCA, ACA and ICA with a free-floating thrombus in the ascending aorta	
31	Encephalopathy and seizure activity in a COVID-19 well controlled HIV patient [37]	CT—head MRI—brain	No abnormality	
32	COVID-19-associated myositis with severe proximal and bulbar weakness [38]	MRI—brain	Extensive edema and enhancement suggestive of inflammatory myopathy Central nonenhancement in the vastus medialis, consistent with myonecrosis	
33	Evolution and resolution of brain involvement associated with SARS- CoV2 infection: a close clinical—paraclinical follow up study of a case [39]	CT—head MRI—brain	High signal abnormalities in B/L pons, thalami, and medial temporal lobes	
34	First case of focal epilepsy associated with SARS-coronavirus-2 [40]	CTA MRI—brain	Proximal left ICA plaques with focal calcification Dilated ventricular system with a prominent and patent aqueduct of Sylvius	
35	First case of SARS-COV-2 sequencing in cerebrospinal fluid of a patient with suspected demyelinating disease [41]	MRI—brain	No abnormality	
36	First motor seizure as presenting symptom of SARS-CoV-2 infection [42]	CT—head	No abnormality	
37	Focal EEG changes indicating critical illness associated cerebral microbleeds in a COVID-19 patient [43]	MRI—brain	Focal injury without encephalopathy Diffuse microbleeds in B/L juxtacortical white matter, corpus callosum, and internal capsule	
38	Fulminant cerebral edema as a lethal manifestation of COVID-19 [44]	CT—head	Extensive vasogenic edema and herniation of temporal lobes toward the brain stem with obliteration of basal cerebral cisterns, multiple juxtacortical microbleeds, which may be compatible with venous hemorrhagic infarction, effacement of ventricles and peripheral sulci and gyri	
39	Intracranial hemorrhage in a young COVID-19 patient [45]	CT—head	Large, multiloculated right ICH associated with vasogenic edema; uncal and sub-falcine herniation without an underlying ischemic stroke	
40	Ischemic stroke associated with novel coronavirus 2019: a report of three cases [46]	CT—head	Case 1. Low-density lesion at right cerebellar suggestive of acute ischemic stroke Case 2. Attenuation and effacement at the right hemisphere around the Sylvian fissure Case 3. Hypo-density at left basal ganglion	
41	Locked-in with COVID-19 [47]	MRI—brain MRA	Numerous foci of restricted diffusion within the pons, (correlating with FLAIR signal abnormality) consistent with acute pontine ischemic infarcts Decreased flow in distal right vertebral artery with a patent basilar artery	
42	Macrothrombosis and stroke in patients with mild COVID-19 infection [48]	CT—head MRI—brain	Nonocclusive thrombus in the right common carotid artery, extending into the ICA Acute stroke in the territory of the right MCA	
43	Malignant cerebral ischemia in a COVID-19 infected patient: case review and histopathological findings [49]	CT—head	Large right MCA infarct	
44	Multiple sclerosis following SARS-CoV-2 infection [50]	MRI—brain	Supratentorial periventricular demyelinating lesions in right occipital lobe and left temporal	
45	Necessity of brain imaging in COVID-19 infected patients presenting with acute neurological deficits [51]	CT—head	Case 1—B/L subacute infarcts, basilar cistern effacement, a left-to-right midline shift, intraparenchymal hemorrhage, sub-falcine, and uncal herniation Case 2—Pre-op - large volume hemorrhage within the right temporal and parietal lobes, surrounding edema, midline shift, uncal herniation, and entrapment of the temporal horns. Post-op—right-sided craniectomy and anterior temporal lobectomy—improvement in overall mass effect	
46	Neuralgic amyotrophy following infection with SARS-CoV-2 [52]	MRI—brain	Edema and inflammatory contrast enhancement of the right distal median nerve Minor right C5-C6 disk protrusion without nerve root impingement, and mild T2-signal increase of the ipsilateral C7-C8 roots, suggestive of proximal edema	
47	Neurological manifestations in critically ill patients with COVID-19: a retrospective study [53]	CT—head	Low density lesions in the following: Case 1. B/L parietal and frontal lobes, right occipital lobe Case 2. Left hemisphere, B/L temporal, and occipital lobes Case 3. B/L parietal and frontal lobes Case 4. Right hemisphere Case 5. Left midbrain Case 6. Right side of the periventricular area	
48	Novel coronavirus (COVID-19)-associated Guillain-BarrÃ© syndrome: case report [54]	MRI—spine	No evidence of myelopathy or radiculopathy	
49	Olfactory gyrus intracerebral hemorrhage in a patient with COVID-19 infection [55]	CT—head MRI—brain	Right olfactory gyrus ICH with surrounding edema, with no evidence of soft tissue injury or cerebral contusion	
50	Orbitofrontal involvement in a neuroCOVID-19 patient [56]	MRI—brain	Hyperintensity of the right orbital prefrontal cortex adjacent to the olfactory bulb, which seemed to spread toward the right caudate nucleus and mesial prefrontal cortex	
51	Posterior reversible encephalopathy syndrome (PRES): another imaging manifestation of COVID-19 [57]	CT—head MRI—brain	Symmetric hypoattenuation of the external capsules and posterior subcortical cerebral white matter Hyperintensity with increased diffusion in the internal and external capsules, subcortical, deep cerebral, and cerebellar white matter	
52	Prolonged confusional state as first manifestation of COVID-19 [6]	CT—head	Mild chronic small vessel ischemic changes	
53	Reversible cerebral vasoconstriction syndrome and dissection in the setting of COVID-19 infection [58]	CT—head	B/L convexity SAH Left vertebral artery dissection	
54	Reversible encephalopathy syndrome (PRES) in a COVID-19 patient [59]	CT—head CTA MRI—brain	Posterior frontal and temporo-parieto-occipital symmetrical B/L hypodensity of the subcortical white matter, and a small left occipital parenchymal hemorrhage Absence of vascular malformation and alterations of posterior circle vessel caliber- suggestive of vasoconstriction mechanism Onset of right temporal hypodensity, correlated to hemorrhagic process	
55	SARS-CoV-2-associated Guillain-BarrÃ© syndrome with dysautonomia [60]	CT—head	No abnormalities	
56	Severe headache as the sole presenting symptom of COVID-19 pneumonia: a case report [61]	MRI—brain MRA	Nonspecific white matter hyperintensities Normal MRA	
57	Steroid-responsive encephalitis in coronavirus disease 2019 [62]	CT—head MRI—brain	No abnormalities	
58	Stroke and COVID19: not only a large-vessel disease [63]	CTA MRI—brain	Small cortical acute ischemic lesions in the right pre- and post- central gyrus, without signs of previous ischemic lesions and hemorrhagic infarction	
59	Stroke in patients with SARS-CoV-2 infection: case series [64]	CT—head MRI—brain	Case 1—CT showed numerous hypodense lesions involving different cortical and subcortical regions of B/L cerebral hemispheres Case 2—Ischemic lesion involving the frontal lobe on the right side; Occlusion of the right pericallosal artery; multiple, B/L supratentorial and infra-tentorial ischemic lesions. Case 3—Small hypodense area in the right thalamus of presumed ischemic origin Case 4—Focal T2-FLAIR HI lesion in the left precentral gyrus with a bright signal on DWI sequence, and mild post-contrast enhancement of the head of right caudate nucleus Case 5—Large cerebellar hemorrhage compressing the brainstem and 4th ventricle causing a subsequent obstructive hydrocephalus Case 6—Diffuse cerebral edema with loss of normal gray—white matter differentiation and obliteration of CSF spaces; large right frontal hemorrhage with other smaller hemorrhages and a bright spot within the sagittal sinus suspected for dural sinus thrombosis	
60	Subcortical myoclonus in COVID-19: comprehensive evaluation of a patient [65]	MRI—brain	Cerebral small-vessel disease of moderate severity	
61	Thalamic perforating artery stroke on computed tomography perfusion in a patient with coronavirus disease 2019 [66]	CT—head MRI—brain	Small focal hypoperfusion in the paramedian perforating vascular territory supplying the left medial thalamus 2 punctate acute ischemic lesions in each cerebellar hemisphere	
62	Two patients with acute meningoencephalitis concomitant with SARS-CoV-2 infection [67]	MRI—brain	Normal	
63	COVID-19 is associated with an unusual pattern of brain microbleeds in critically ill patients [68]	MRI—brain	Microbleeds in unusual distribution, particularly involving the anterior/posterior limbs of internal capsule (five patients), middle cerebellar peduncles (5/9 patients), and the corpus callosum

**Fig. 3:**
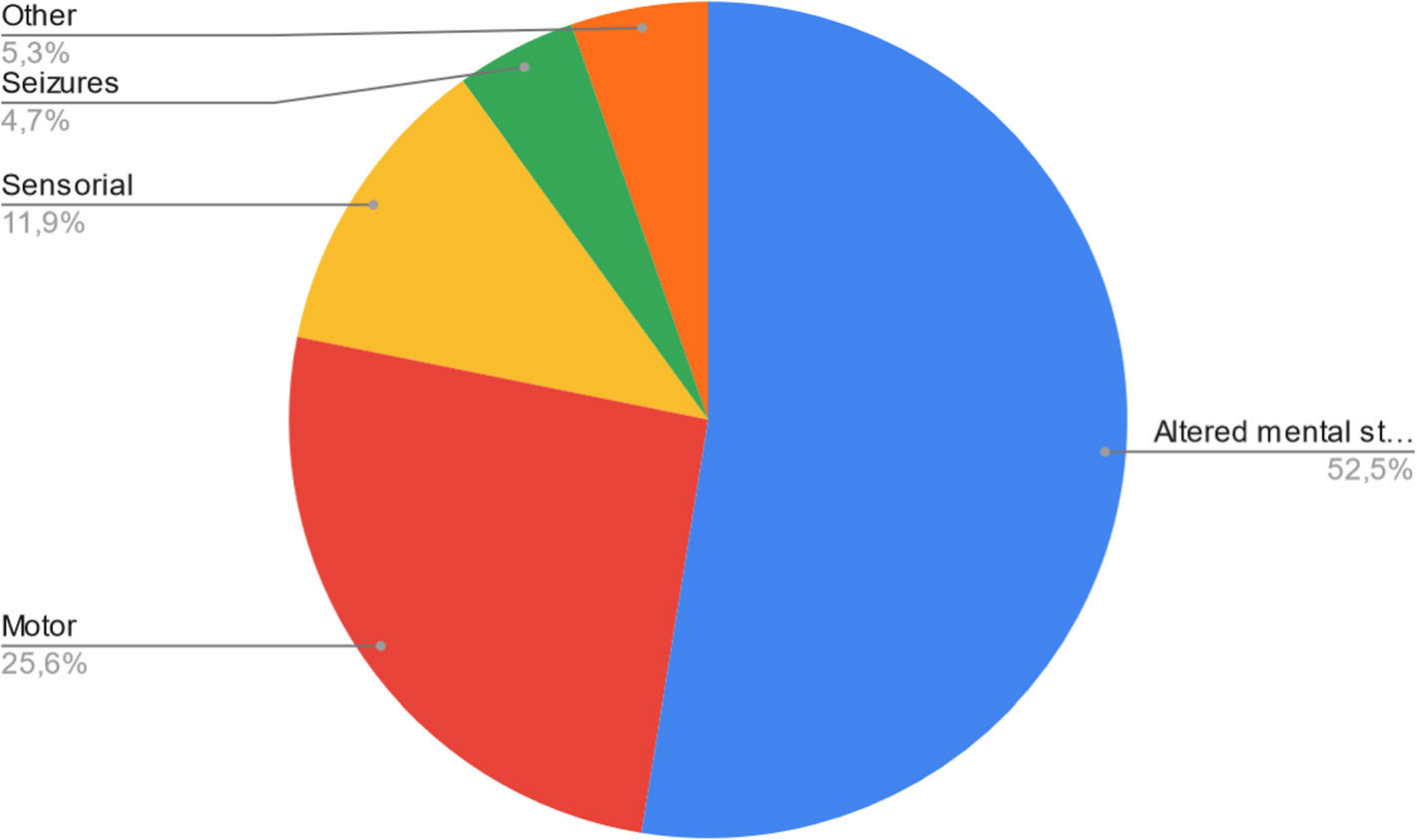
Neurological manifestations in patients infected with SARS-CoV2

**Fig. 4:**
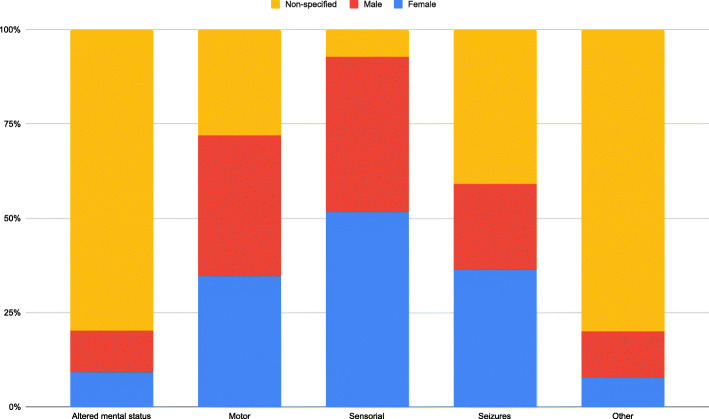
Gender wise allocation of neurological manifestations in patients with COVID-19

Out of the 157 distinct neurological manifestations presented in the 63 articles (Table 2), we were able to identify 5 possible groups. Patients were only included once per group. In order of prevalence: altered mental status (52.5%), sensory alterations (19.7%), motor alterations (17.7%), others (5.5%), and seizures (4.6%) (Fig. 3). Certain articles with a larger patient population did not specify its prevalence for the different neurological manifestations. The only group with a female predominance was sensory alterations (51.7%). No group had a defined male predominance. Altered mental status and others had a greater representation of un-specified sex (79.8% and 80% respectively) (Fig. 4).

**Table 2:** General signs and symptoms, and associated neurological manifestations reported in the studies on COVID-19 infected patients

	Article name	Article type	*N* = no. of patients	Age/sex	General signs and symptoms	Neurological manifestations
1	A case of COVID-19 respiratory illness with subsequent seizure and hemiparesis [9]	Case report	1	38-year-old male	Progressive cough Fever Dyspnea	Generalized tonic—clonic seizure (GTCS) Left-sided hemiplegia Decreased right side spontaneous movements
2	A case series of devastating intracranial hemorrhage during venovenous extracorporeal membrane oxygenation for COVID-19 [3]	Case series	4	Mean age—50.7 years		Anisocoria Gaze defect Altered mental status (AMS) Agitation
3	A first case of meningitis/encephalitis associated with SARS-coronavirus-2 [10]	Case report	1	24-year-old male	Headache Generalized fatigue Fever and sore throat	Neck stiffness Transient generalized seizures Glasgow coma scale (GCS)—6/15
4	Acute abducens nerve palsy in a patient with the novel coronavirus disease (COVID-19) [11]	Case report	1	32-year-old male	Fever and cough Diarrhea Fatigue	Diplopia (acute, binocular, horizontal)
5	Acute disseminated encephalomyelitis after SARS-CoV-2 infection [12]	Case report	1	64-year-old female	Influenza-like syndrome	Anosmia, ageusia B/L vision impairment Right leg sensory deficit
6	Acute myelitis as a neurological complication of COVID-19: a case report and MRI findings [13]	Case report	1	32-year-old male	Flu-like symptoms	Urinary retention B/L lower limb weakness
7	Acute polyradiculoneuritis with locked-in syndrome in a patient with COVID-19 [14]	Letter to the editor	1	51-year-old male	Flu-like symptoms	Progressive upper and lower limb weakness Acral paresthesia
8	Acute profound sensorineural hearing loss after COVID-19 pneumonia [15]	Correspondence (case report)	1	60-year-old male	Fever with cough	Sensorineural hearing loss
9	Basal ganglia involvement and altered mental status: a unique neurological manifestation of coronavirus disease 2019 [16]	Case report	1	54-year-old female	Low-grade fever Cough	AMS GCS- 10/15
10	Bilateral posterior cerebral artery territory infarction in a SARS-Cov-2 infected patient: discussion about an unusual case [17]	Case report	1	51-year-old male	Cough Diarrhea	Headache Dysgeusia Abrupt cortical blindness Disorientation
11	Bilateral trochlear nerve palsy due to cerebral vasculitis related to COVID-19 infection [18]	Case report	1	69-year-old male	Fever Abdominal pain Left posterior chest pain	Binocular diplopia Severe stabbing occipital headache Bilateral paresis of CN IV
12	Cerebral microhemorrhage and purpuric rash in COVID-19: The case for a secondary microangiopathy [19]	Case report	1	69-year-old male	Dyspnea, cough Diarrhea Fever Diffuse rash	Deterioration of mental status
13	Cerebral nervous system vasculitis in a COVID-19 patient with pneumonia [20]	Case report	1	64-year-old male	Fever Cough	Tetraplegia and B/L mute plantar response GCS- 6/15
14	Cerebral venous thrombosis: a typical presentation of COVID-19 in the young [21]	Case report	1	25-year-old female	Cough Low-grade fever Mild shortness of breath	GTCS with post-ictal confusion Decreased level of arousal Global aphasia Right facial nerve palsy B/L CN VI palsy
15	Coexistence of COVID-19 and acute ischemic stroke report of four cases [22]	Case report	4	45-year-old female 67-year-old female 72-year-old male 77-year-old male	Fever Cough Shortness of breath	Left facial paresis Dysarthria Hemiparesis Loss of consciousness Mild ataxia Left hemi-hypoesthesia
16	Concomitant neurological symptoms observed in a patient diagnosed with coronavirus disease 2019 [23]	Case report	1	64-year-old male	Fever with mild cough Insomnia Muscle soreness	Poor mental state B/L ankle clonus, Left Babinski sign + Neck Stiffness with Brudzinski sign +
17	Coronavirus 2019 (COVID-19)-associated encephalopathies and cerebrovascular disease: the New Orleans experience [24]	Retrospective cohort study	27	Mean age—59.8 years		Altered mental status Headache Dysgeusia Gaze deviation Focal deficits Hemiparesis/hemiplegia
18	COVID-19 presenting as stroke [25]	Case series	4	73-year-old male 83-year-old male 80-year-old female 88-year-old female	Fever Respiratory distress Nausea/vomiting Reduced oral intake	Altered mental status Facial drop Slurred speech Left-sided hemiparesis Right-arm weakness Word-finding difficulty
19	COVID-19 presenting with seizures [26]	Case report	1	72-year-old male	Weakness, lightheadedness after a hypoglycemic episode Shortness of breath	AMS Multiple episodes of tonic—clonic movements of upper and lower limbs
20	COVID-19 related neuroimaging findings: a signal of thromboembolic complications and a strong prognostic marker of poor patient outcome [27]	Retrospective cohort study	454	Median age—64 years		AMS/delirium (37.6%) Stroke (17.3%) Mechanical fall/ trauma (25.5%) Syncope (4%) Headache (3.8%) Dizziness (2.8%) Seizure (2.1%) Ataxia (1.4%)
21	COVID-19-associated encephalopathy with fulminant cerebral vasoconstriction: CT and MRI findings [28]	Case report	1	50-year-old male	Fatigue Nausea Vomiting	Severe headache Worsening lethargy Fixed mydriasis with deviation toward the left
22	COVID-19-associated encephalopathy: neurological manifestation of COVID-19 [29]	Case report	1	43-year-old male	Fever, dry cough Generalized weakness	Decreased level of consciousness GCS- 3/15
23	COVID-19-associated ophthalmoparesis and hypothalamic involvement [30]	Case report	2	60-year-old female 35-year-old female	Patient 1. Fever Nausea Cough Patient 2. History of vomiting	Patient 1. Right CN VI palsy Hyposmia Right hemi-cranial headache Diplopia Patient 2. Diplopia Paresthesia Decreased arousal Disorientation Episodic memory deficits B/L CN VI palsy Mild paraparesis
24	COVID-19-associated pulmonary and cerebral thromboembolic disease [31]	Case report	1	79-year-old female		Aphasia Left hemiparesis
25	COVID-19-related acute necrotizing encephalopathy with brain stem involvement in a patient with aplastic anemia [32]	Case report	1	59-year-old female	Sore throat Shortness of breath Myalgia Vomiting	Episodes of vacant staring Speech arrest Flexion of both shoulders GTCS with post-ictal reduced consciousness
26	COVID-19-related strokes in adults below 55 years of age: a case series [33]	Case series	6	33-year-old female 39-year-old male 40-year-old male 47-year-old female 49-year-old female 53-year-old male	Cough Dyspnea Myalgia Lethargy Headache	Altered consciousness Global aphasia Hemiplegia Left side weakness Homonymous hemianopia Sensory deficit Dysarthria
27	COVID-19-associated encephalitis mimicking glial tumor [34]	Case report	1	35-year-old female	Headache Nausea	Drug-refractory seizures Dizziness
28	De novo status epilepticus in patients with COVID-19 [35]	Case series	2	49-year-old female 73-year-old female	Patient 1. None Patient 2. Shortness of breath Lower limb edema	Patient 1. B/L tonic clonic seizures Altered mental status Patient 2. Face and arm myoclonus Altered mental status
29	Delirium as a presenting feature in COVID-19: neuroinvasive infection or autoimmune encephalopathy? [36]	Case report (letter to the editor)	2	46-year-old male 79-year-old female		Patient 1. Status epilepticus Acute hypoactive delirium Disinhibition Headache Patient 2. Generalized seizure Dysphasia Impaired orientation, attention and memory
30	Emergency room neurology in times of COVID-19: malignant ischaemic stroke and SARS-CoV-2 infection [7]	Case report	1	36-year-old female	Unconsciousness	Global aphasia Right hemiplegia
31	Encephalopathy and seizure activity in a COVID-19 well controlled HIV patient [37]	Case report	1	41-year-old male	Abdominal pain Intractable vomiting Dry cough Intermittent fever	Confusion and agitation GTCS Left-sided ptosis
32	COVID-19-associated myositis with severe proximal and bulbar weakness [38]	Case report (letter to the editor)	1	58-year-old female	Cough Dyspnea Myalgia with severe generalized weakness Dysphagia Odynophagia	Proximal bulbar weakness Bilateral ptosis Facial weakness Hypernasal dysarthria Profound symmetric proximal limb weakness
33	Evolution and resolution of brain involvement associated with SARS-CoV2 infection: a close clinical—paraclinical follow up study of a case [39]	Case report	1	39-year-old female	Fever with dry cough Myalgias and anorexia	Decline in consciousness Multiple episodes of GTCS
34	First case of focal epilepsy associated with SARS-coronavirus-2 [40]	Case report	1	73-year-old female	Fatigue Dry cough Back pain	Painful muscle stiffening and twitching in the left leg and arm (focal seizure)
35	First case of SARS-COV-2 sequencing in cerebrospinal fluid of a patient with suspected demyelinating disease [41]	Case report	1	42-year-old female	Mild respiratory symptoms	Paresthesia and hypoesthesia in left upper limb, left hemithorax, and hemiface
36	First motor seizure as presenting symptom of SARS-CoV-2 infection [42]	Case report	1	54-year-old male	Conjunctivitis Fever	Clonic movements in the right arm Loss of consciousness
37	Focal EEG changes indicating critical illness associated cerebral microbleeds in a COVID-19 patient [43]	Case report	1	56-year-old female	Cough Fever	Agitation Impaired cognition and vigilance Executive dysfunction
38	Fulminant cerebral edema as a lethal manifestation of COVID-19 [44]	Case report	1	57-year-old male	Fatigue and fever Dyspnea Nausea/vomiting Diarrhea	Dilated and nonreactive pupils Absent brain stem reflexes
39	Intracranial hemorrhage in a young COVID-19 patient [45]	Case report	1	42-year-old male	Severe cough Fever (103Â°F) Dyspnea Pleuritic chest pain	U/L pupillary changes- progressed to B/L fixed and dilated pupils Loss of all brain stem reflexes
40	Ischemic stroke associated with novel coronavirus 2019: a report of three cases [46]	Case reports	3	88-year-old female 85-year-old female 55-year-old male	Fever Dry cough Asthenia	Ataxia Dysarthria Impaired orientation Drowsiness Peripheral/central facial paresis Limb weakness Impaired memory Acute hemiplegia Broca’s aphasia
41	Locked-in with COVID-19 [47]	Case report	1	25-year-old female	Cough Shortness of breath Fever Malaise	Unable to exhibit motor functions Only able to follow commands through horizontal eye movement and eye blinking B/L Babinski sign +
42	Macrothrombosis and stroke in patients with mild COVID-19 infection [48]	Case report	3	33-year-old female 77-year-old female 55-year-old male	Cough	Patient 1—Left sided hemiplagia with hemisensory loss Patient 2—Sudden onset aphasia with left side hemiparesis Patient 3—Left sided weakness
43	Malignant cerebral ischemia in a COVID-19 infected patient: case review and histopathological findings [49]	Case report	1	48-year-old male	Dyspnea Cough	Left-sided hemiplegia and neglect Speech abnormalities
44	Multiple sclerosis following SARS-CoV-2 infection [50]	Case report	1	29-year-old female	Anosmia, dysgeusia Asthenia	Reduced visual acuity in right eye Eye movements associated with increased retro-ocular pain and color desaturation Pyramidal tract dysfunction
45	Necessity of brain imaging in COVID-19 infected patients presenting with acute neurological deficits [51]	Case study	2	37-year-old female 47-year-old female	Patient 1. Fever, cough Shortness of breath Patient 2. Lethargy	AMS
46	Neuralgic amyotrophy following infection with SARS-CoV-2 [52]	Case report	1	52-year-old male	Rhinorrhea Headache	Persistent severe pain in the right shoulder aggravated by arm extension with gradual shift to forearm and hand Paresthesia of index and long fingers Progressive weakness of right hand
47	Neurological manifestations in critically ill patients with COVID-19: a retrospective study [53]	Retrospective case series	7	Mean age—66 Â± 11.1 years	Fever Cough Myalgia Fatigue Headache Dizziness	Delirium Acute ischemic stroke Intracerebral hemorrhage Hypoxic-ischemic brain injury Flaccid paralysis
48	Novel coronavirus (COVID-19)-associated Guillain-BarrÃ© syndrome: case report [54]	Case report	1	54-year-old male	Rhinorrhea Odynophagia Fevers, chills, and night sweats	Ascending limb weakness and numbness Quadriparesis Facial diplegia Mild ophthalmoparesis
49	Olfactory gyrus intracerebral hemorrhage in a patient with COVID-19 infection [55]	Case report	1	72-year-old male	Anosmia Loss of appetite	Focal onset status epilepticus with Todd’s paralysis
50	Orbitofrontal involvement in a neuroCOVID-19 patient [56]	Case report	1	69-year-old male	Cough Fever	Anosmia Status epilepticus
51	Posterior reversible encephalopathy syndrome (PRES): another imaging manifestation of COVID-19 [57]	Case report	1	59-year-old male	Fever Dyspnea	Encephalopathy
52	Prolonged confusional state as first manifestation of COVID-19 [6]	Case report	1	77-year-old male	Lethargy	Prolonged confusion
53	Reversible cerebral vasoconstriction syndrome and dissection in the setting of COVID-19 infection [58]	Case report	1	30s female	Severe cough	Severe thunderclap headache
54	Reversible encephalopathy syndrome (PRES) in a COVID-19 patient [59]	Case report	1	64-year-old female	Fever Dyspnea	Drowsiness Blurred vision AMS Decreased left nasolabial fold Decreased strength and tone in B/L lower limbs DTRs decreased
55	SARS-CoV-2-associated Guillain-BarrÃ© syndrome with dysautonomia [60]	Letter to the editor	1	72-year-old male	Mild diarrhea Anorexia Chills	Symmetric paresthesia Ascending appendicular weakness Tendon reflexes- absent Diminished sensation to light touch SIADH and Dysautonomia
56	Severe headache as the sole presenting symptom of COVID-19 pneumonia: a case report [61]	Case reports and case series	1	76-year-old female		Severe generalized headache Neck pain
57	Steroid-responsive encephalitis in coronavirus disease 2019 [62]	Case report	1	60-year-old male	Fever Cough Asthenia	Cognitive fluctuations Severe akinetic syndrome associated with mutism Palmomental and glabella reflexes + Moderate nuchal rigidity
58	Stroke and COVID-19: not only a large-vessel disease [63]	Case report	1	49-year-old female		Dysarthria Left side hemiparesis, hemianesthesia, and facial weakness
59	Stroke in patients with SARS-CoV-2 infection: case series [64]	Retrospective observational case series	6	Median age—69 years	Fever Cough Dyspnea	Left-sided hemiparesis B/L fixed and dilated pupils Loss of consciousness Confusion Behavioral abnormalities
60	Subcortical myoclonus in COVID-19: comprehensive evaluation of a patient [65]	Case report	1	58-year-old male	Fever Cough Dyspnea	Myoclonus elicited by action and tactile stimuli predominant in right proximal inferior limb muscles
61	Thalamic perforating artery stroke on computed tomography perfusion in a patient with coronavirus disease 2019 [66]	Case report	1	50-year-old male	Bilateral pneumonia	Sudden right facial palsy Mild Right limb weakness
62	Two patients with acute meningoencephalitis concomitant with SARS-CoV-2 infection [67]	Case report	2	64-year-old female 67-year-old female	Flu-like symptoms	Tonic clonic seizures Headache Psychotic symptoms Disorientation with motor perseverations with B/L grasping Aggressiveness Left hemianopia Sensory hemineglect
63	COVID-19 is associated with an unusual pattern of brain microbleeds in critically ill patients [68]	Case series	9	Mean age—67.7 years	Fever Cough Dyspnea	Delayed recovery of consciousness Psychomotor agitation Confusion

## Discussion

Since the outbreak of the SARS-CoV2 virus in December 2019, the majority of research has been centered around respiratory pathogenesis and manifestations of the virus. However, recent focus has shifted toward its invasive nature and complications in the nervous system. There has been a surge in the number of cases documenting the nervous system involvement in COVID-19 positive patients with minimal respiratory involvement. Some studies reported absence of SARS-CoV-2 RNA in the nasal and throat swabs even though it was found to be present in the cerebrospinal fluid upon further investigations [10]. However, our understanding of the pathophysiology behind such neurological manifestations and the data on neuroimaging still remains limited.

### Pathogenesis

Currently, there are 4 mechanisms of neuro-invasion that have been hypothesized.

### Receptor modulation

The body has a traditional angiotensin-converting enzyme (ACE) in lung capillaries which is a part of the renin-angiotensin-aldosterone system (RAAS) and is involved in regulating blood pressure. COVID-19 is known to use ACE2 receptors, present in the endothelium of the heart, kidneys, and alveolar cells, especially alveolar type 2 (AT2), for cell entry. Binding to these receptors, the virus hampers the body’s natural mechanism of decreasing blood pressure thus increasing the likelihood of intracranial hemorrhages and stroke [69–71]. The neurons and glial cells are known to have ACE2 receptors, possibly explaining the neurotropism of the virus [72]. The mechanism of entry hypothesized is that the spikes present on the virus might link with ACE2 on the capillary endothelium, damaging the blood-brain barrier (BBB) and thus gaining entry into CNS [71]. The two areas are involved in the central regulation of respiration—nucleus of the tractus solitarius and ventrolateral medulla also express ACE2 receptors.

### Trans-cribrial transmission

The anosmia in many cases points toward viral entry via olfactory bulb and across the cribriform plate [71]. This mechanism has been linked with murine experiments which led to the detection of the virus in the midbrain, basal ganglia, infralimbic cortex, and the piriform via intranasal inoculation of COVID-19 [69, 73]. SARS-CoV-2 may use ACE2 or trans-membrane protease serine 2 (TMPRSS2) receptors to infect olfactory receptor neurons in the olfactory epithelium [74].

### Blood-brain barrier spread

Prior research of SARS-CoV and MERS has shown that cytokines like tumor necrosis factor (TNF-α) and interleukins (IL-6 and IL-1) led to direct death of neurons in the respiratory center in the medulla [73, 75]. The prolific response of the immune system leads to an enormous release of these cytokines and chemokines. They lead to increased permeability and breakdown of the BBB resulting in increased entry of leukocytes. They can also precipitate glutamate receptor-induced neuronal hyperexcitability which may be the reason behind acute seizures linked with the virus. Furthermore, hyperinflammatory and immune responses can result in cytokine storm syndrome which is a severe manifestation of COVID-19 [72].

### Trans-synaptic transmission

The entry of the virus into CNS through the peripheral nerves is another hypothesized secondary pathway. The alveoli in the lungs have sensory innervations that detect changes in O_2_ and CO_2_. These pathways run-up to the respiratory centers in the brainstem and send signals to the pre-synapses there. Porcine hepatitis E virus studies depict a similar pathway of transmission and since HEV is almost homologous to hCoV-OC43^2^, a close relative of SARS-CoV-2, it might be the same case here [76].

The neuropathological mechanisms reported to play a role in the development of neurological disorders in COVID-19 are—hypoxic brain injury and immune-mediated damage. The hypoxic brain injury is believed to be due to the alveolar gas exchange disorders caused by proliferation of virus in the alveolar cells [71]. As mentioned above, severe immune response resulting in a cytokine storm can also lead to the development of neurological manifestations [72].

### Neuro-radiological manifestations

About 17.85% patients who underwent neuroimaging were found to be having ischemic changes suggestive of a stroke. Rajan Jain [27] and colleagues found that the inpatient COVID-19 positive population with stroke had a poor outcome. Similarly, in a systematic review by Sebastian Fredman [77] and colleagues, mortality rate of 45% was reported in the admitted COVID-19 positive patients affected with ischemic stroke. Large vessel involvement was found to be the most common, particularly the MCA. The association of COVID-19 and cerebrovascular disease has been well established but it is still unclear whether this is a de novo occurrence or a complication of already existing atheromatous plaques [78]. The role of stenotic lesions resulting in ischemic changes is also unclear. Hemorrhagic changes were found to be the second most common positive imaging finding particularly involving the corpus callosum and subcortical parenchyma. Aikaterini Fitsiori [68] and colleagues reported that COVID-19 or its treatment may cause unusual microbleeds, predominantly affecting the corpus callosum. All these patients were suffering from severe or moderate acute respiratory distress. This could be due to microangiopathic changes resulting from the cytokine-induced pathogenesis discussed above. Simon Pao [79] and colleagues concluded that ischemic changes were seen in both mild and severe infections whereas hemorrhagic changes were more prevalent in severely affected patients.

### Neurological findings

In this study, we observe that COVID-19 patients presented with a variety of neurological complications. In our review, the most prevalent finding has been altered mental status (52.5%). Among the earliest articles about COVID-19 by Mao [5] and colleagues was a retrospective study that showed that 36.4% of patients presented with nervous system abnormalities, and among them, patients who had severe disease were more vulnerable to acute cerebrovascular disease and altered consciousness. The neurotropism of the virus leading to inflammation in the CNS may be a cause of altered mental status. Macrophages and microglia which proliferate to the areas concentrated by viral antigen have shown to cause demyelination leading to memory and cognitive deficits. This was observed in a murine study conducted with several strains of the virus [80, 81]. Nepal G [80]. and colleagues mention the importance of early identification of altered mental status in SARS-CoV-2 patients to check for a possible reversible cause leading to its early management. Confusion, agitation, drowsiness, lethargy, and psychotic symptoms were some of the most commonly observed subsets of symptoms included in altered mental status (Table 2).

Stroke has been observed to be the most frequent finding in neuroimaging of patients affected by COVID-19. A peculiar thing about COVID-19 related strokes is that they can be found in younger patients as observed in a case series by Ashrafi [33] which explores this association in patients younger than the age of 55, where the youngest patient, a 33-year-old, was without any previous comorbidities. Several studies have mentioned the prothrombotic and inflammatory nature of COVID-19, and some reports mention stroke symptoms being the first presentation in many cases. Lee SG [82] and Spence JD [83] mention that about 20-55% of SARS-CoV-2 patients exhibited laboratory values indicating coagulopathies. The prevalence of ischemic strokes is slightly higher than that of hemorrhagic strokes as seen in a 6-patient case series by Morassi [64] where 4 were affected by ischemic stroke and 2 by hemorrhagic. Other frequently seen manifestations include paralysis, headaches, and altered speech.

As far as we know, this is the only study with documentation of reports published until August 2020 which is based on the nervous system involvement and neuroradiological findings of COVID-19 patients. The limitations of our study were that a subset of reported neurological or neuroimaging findings in severely ill and elderly patients may be incidental. The radiological findings might have been susceptible to clinical bias hence it is difficult to standardize them. Radiological imaging presumably is performed selectively on those presenting with notable neurological involvement, leaving out the probable findings in those diseases which are milder in nature, as routine imaging may increase the risk of transmission of the virus. Our study only included articles published in the English language.

## Conclusion

In the past few months of the global pandemic, the connection between COVID-19 and neurological manifestations has been growing substantially. Having strong knowledge about such associations will prove to be instrumental in early detection, isolation, and care of patients who present with unusual neurologic symptoms, especially during the ongoing pandemic. Focus on long-term neurologic sequelae and neuroimaging findings is necessary to further the research on the neurotropic involvement of SARS-CoV-2.

## Data Availability

The authors declare that the data supporting the findings of this study are available within the article [and its supplementary information files].

## Abbreviations

ACE: Angiotensin-converting enzyme
ACE2: Angiotensin-converting enzyme 2
AT2: Alveolar type 2 cells
BBB: Blood-brain barrier
CNS: Central nervous system
CT: Computed tomography
HEV: Hepatitis E virus
ICA: Internal carotid artery
IL-1: Interleukin 1
IL-6: Interleukin 6
MCA: Middle cerebral artery
MRA: Magnetic resonance angiography
MRI: Magnetic resonance imaging
RAAS: Renin angiotensin aldosterone system
SARS-CoV2: Severe acute respiratory syndrome-coronavirus 2
TMPRSS2: Transmembrane protease serine 2
TNF: Tumor necrosis factor
